# Microbial Profile of Fresh and Semicooked Nile Tilapia (*Oreochromis niloticus*) and Hygienic Practice of Fish Handlers in Hawassa, Ethiopia

**DOI:** 10.1155/2023/5866719

**Published:** 2023-11-14

**Authors:** Fasika Admasu, Abraham Mikru, Kassaye Balkew, Million Adane

**Affiliations:** ^1^Cecilia Comprehensive Secondary School, Hawassa, Ethiopia; ^2^Department of Biology, Hawassa University, P.O. Box 5, Hawassa, Ethiopia; ^3^Department of Aquaculture and Fishery Technology, Hawassa University, P.O. Box 05, Hawassa, Ethiopia; ^4^Ethiopian Biodiversity Institute, P.O. Box 30726, Addis Ababa, Ethiopia

## Abstract

Despite its high nutritional quality, fish is a highly perishable food item. This study aimed at assessing the microbial quality and safety of fresh and semicooked Nile tilapia fish fillets and the food safety practices of fish handlers in Hawassa City. The microbial load of 40 for each of raw and semicooked fillet samples was estimated by the standard plate count method, and the dominant flora as well as common bacterial pathogens were identified following phenotypic procedures. Moreover, a survey was conducted to assess the hygienic conditions and food safety practices of 30 fish handlers. The mean microbial load of the raw fillet samples in log_10_CFUg^−1^ was 8.42 for aerobic mesophilic bacteria (AMBC), 2.52 for total coliforms (TCC), and 3.41 for a count of staphylococci (CS). On the other hand, the respective parameters for the semicooked fillets in log_10_CFUg^−1^ were 6.68 (AMBC), 2.52 (TCC), and 3.17 (CS). The mean AMBC of all the fresh raw fillet samples exceeded the recommended maximum permissible limits. The mean SC of raw fillets from three of the eight vendors and one semicooked fillet were at a potentially hazardous level (>4 log units). Moreover, *Salmonella* species were isolated from 30% to 25% of raw and semicooked samples, respectively. The mesophilic bacterial flora of both types of samples was dominated by *Bacillus* species, *Salmonella* species, *E coli*, and *Staphylococcus* species. Most fish handlers did not practice hygienic food handling and lacked basic sanitation amenities like clean water and soap for hand washing. Moreover, nearly all the fish handlers did not have any formal education. These findings call for public health intervention measures like the provision of training in good hygienic practices and certification for fish vendors in the chain.

## 1. Introduction

Fish is an important part of a healthy diet as it provides high-quality protein, and omega-3 fatty acids [1, 2]. Globally, fish contributes about 60% of the world's supply of protein [3]. Owing to its digestibility, fish protein complements diets provided by cereals and legumes that are typically consumed in many developing countries. Fish is also a valuable source of vitamins A and B and iodine [4]. Moreover, a fish diet is known to reduce the risk of heart disease, inflammation, and cancer [5, 6]. Recognition of these nutritional and health benefits provided by the fish diet is such that world per capita fish consumption increased from 9.9 kg in the 1960s to 20.1 kg in 2016 [7].

However, in many African countries, including Ethiopia, fish production and consumption are very low. Being a land-locked country, Ethiopia depends for its fishery on inland water bodies, which include an estimated surface area of 7,334 km^2^ of major lakes and reservoirs, 7,185 km of rivers, and 275 km^2^ of small water bodies [8]. The total annual fish production potential of the country is about 51,481 tons [9]. These estimates did not consider the potential of newly developed reservoirs and dams being constructed. A more realistic estimate of the fishery production potential of the Ethiopian inland water bodies puts it between 89,000 and 99,000 tons per year [10]. The inland capture fishery provides nearly all the fish supplies in Ethiopia, with a barely budding aquaculture supply starting recently [7]. The country's overall per capita fish production is less than 480 g per person per year [7]. However, in areas where there is a regular and sufficient supply, annual fish consumption can exceed 10 kg per person per year [11]. According to Christophe and Damien [8], about 73% of the total fish landed was marketed as fresh in nearby markets, while 26% was transported to distant areas as chilled or frozen, and only 1% of the products were processed in canned, smoked, or dried forms.

Lake Hawassa is the major source of fish supply for the residents of Hawassa City, the administrative center of the Sidama Regional State of Ethiopia (SRSE). The lake has an estimated surface area of about 90 km^2^, with a potential for 600 metric tons of fish production per year [8]. Fishing activity in Hawassa is mainly an artisanal activity operated with basic rafts made of papyrus and wooden canoes, the use of hook lines, and some nets. Among the fish types supplied from the lake, Nile tilapia (*Oreochromis niloticus*) is the most popular one. The main customers are the many restaurants that serve the fish in different recipes, either fried or raw. Fish dishes are special attractions for local and foreign tourists, particularly during lent and normal fasting days (Wednesdays and Fridays). These facts have led to increasing demand and created a lucrative business for mushrooming small-scale fish vendors. Raw fish fillets (“AsaKurt”) and semicooked fish fillets (“AsaLebleb”) are among the most popular fish dishes.

Despite all these positive vibes, there are many concerns, as fish is one of the most perishable food items that deteriorate rapidly and may also serve as a vehicle for the transmission of food-borne pathogens [12, 13]. The spread and persistence of human pathogens in fish and fish products and the formation of hazardous toxins caused by the growth of spoilage microorganisms are major concerns. *Clostridium botulinum,* pathogenic *Vibrio spp.*, *Aeromonas spp*., and *Plesiomonas shigelloides* are examples of human pathogenic bacteria that may be inherently present as part of the fish's normal flora [14–16]. Moreover, contamination of fish from external sources during processing and storage may add pathogenic bacteria like *Listeria monocytogenes, Staphylococcus aureus, Salmonella spp*., *Shigella spp*., *Escherichia coli,* and *Yersinia enterocolitica* [17]. Studies have demonstrated the occurrence of potentially pathogenic and spoilage bacteria in fish, including *Pseudomonas angulluseptica* and *Streptococcus* spp [3].

Several factors may contribute to the microbial deterioration of the quality and safety of fish, including lack of proper transportation facilities, ignorance, poor handling, and processing, among others [18, 19]. Fish production and distribution in Ethiopia are carried out both formally (e.g., through organized cooperatives) and informally by private individuals [20]. According to some estimates, there are collectively about 67,400 fishermen operating among the different inland water bodies in Ethiopia [10]. Updated information about the number of people engaged in the sector for their livelihood is unavailable; nonetheless, one would expect a steady increase over the decade since.

Similar to most developing nations, Ethiopia's fish supply chain primarily follows an informal market system with limited supply chain management for fish quality and a lack of information for price determination along the chain [21, 22]. Under these conditions, the supply chain faces difficulties in maintaining quality and tracing the paths of the products when spoiled or substandard products reach the consumer.

Experiences in other parts of the world have shown that fish and fish products have been involved in various food-borne disease outbreaks and product recalls annually [13]. The outbreak of fishborne disease could affect not only direct consumers but also have a ripple effect on the hotel and tourism industries by tainting the positive image of the country. From an economic perspective, the fisheries subsector contributes very little to the overall GDP in Ethiopia [10]. This would definitely change if spoilage and postharvest losses were minimized by installing good manufacturing and hygienic practices at the various steps along the production and supply chain [19]. The objective of this study was to assess the microbial quality and safety of raw and undercooked Nile tilapia fillets as well as the food safety practices of fish handlers in Hawassa city, southern Ethiopia.

## 2. Materials and Methods

### 2.1. Description of the Study Area

The study was conducted from November 2017 to June 2018 in Hawassa City, the administrative center of the Sidama Regional State of Ethiopia (SRSE). Hawassa is located on the southeastern shores of Lake Hawassa, 275 km south of Addis Ababa, within the geographic coordinates 06°58′–07°14′ N latitudes and 38°22′–38°28′ E longitudes [23]. The city is one of the hotspots of tourist destinations for locals as well as foreigners and consequently hosts dozens of international hotels and resorts. The samples for the study were collected from Lake Hawassa landing sites and its surrounding hotels.

### 2.2. Study Design

A cross-sectional study was conducted based on the laboratory analysis of the microbiological profiles of raw and semicooked Nile tilapia fillet samples collected from randomly selected hotels in Hawassa and lakeside fish vendors. Moreover, a questionnaire survey of randomly selected fish handlers was done to assess the current status of food safety practices.

### 2.3. Sample Collection and Culture Media Preparation

A total of 80 (40 fresh raw and 40 semicooked) Nile tilapia fillets were collected in 20 trips from 10 randomly selected registered hotels and lakeside fish vendors. From each vendor, four fresh, raw, and four semicooked fillet samples were considered. On each trip, Nile tilapia fillet (one fresh raw and one semicooked) samples were collected from one registered hotel and one lakeside fish vendor. All samples were collected aseptically using a sterile stainless-steel box, labeled with the type of sample, place, date of sampling, given an identification code, and transported to Hawassa University Microbiology Laboratory in an icebox containing ice packs. Upon arrival, the samples were stored in a refrigerator until processing within two hours of collection. All media used in this study were prepared according to the instructions of the manufacturers (Himedia Laboratories, India).

### 2.4. Preparation of Decimal Dilution of the Fish Fillet Samples

Each sample was prepared following the protocol for the standard plate count method [24]. Briefly, each cutlet of the fillet sample was well mixed, and 10 g pieces were aseptically transferred using sterile forceps and a dissecting scalpel into a labeled bottle containing 90 mL of sterile buffered peptone water as a diluent to make a tenfold dilution. The bottle was vigorously agitated manually and by a vortex mixer for 5 minutes to release the attached microbes from the fillet pieces. Further, tenfold serial dilutions were done up to 10^−7^ by transfer of 1 mL aliquots aseptically using a sterile micropipette to test tubes containing 9 mL buffered peptone water [25]. Vortex mixing was done between each transfer into the tubes to ensure uniform homogenization. The pH of the sample was measured from the 10^−1^ dilution using a digital pH meter (digital pH, model 181).

### 2.5. Enumeration of the Microbial Load of the Fillet Samples

For aerobic mesophilic bacterial count (AMBC), 0.1 mL aliquots from 10^−6^ and 10^−7^ dilution tubes were transferred on plate count agar (PCA, HiMedia) using a sterile micropipette and spread-plated with a sterile bent glass rod in duplicates. Then, the inoculated plates were incubated at 37°C for 48 hrs. For the enumeration of total coliforms (TCC) and *Staphylococcus aureus*, 0.1 mL aliquots from dilution factors of 10^−2^ and 10^−3^ were spread plated on violet red bile agar (VRBA, Hi Media) plates and manitol salt agar (MSA, HiMedia) plates in duplicates, respectively. Morphologically typical colonies (pink colonies on VRBA and golden yellow colonies on MSA) were counted from countable plates using a colony counter with a magnifying glass. Plates having between 25 and 250 colonies were considered to calculate the average load in colony-forming units per gram of fish fillet samples (CFUg^−1^) [26].

### 2.6. Identification of Dominant Bacteria and Common Pathogens

For aerobic mesophilic bacteria, five to ten morphologically distinct colonies were picked separately from countable PCA plates and purified by repeated subculturing on nutrient agar plates, and their colony morphology was noted. Purified isolates were maintained in 20% glycerol cryo-preservative vials (containing 800 *μ*l sterile nutrient broth and 200 *μ*L of sterile glycerol) and stored in a deep freezer at −20°C until further biochemical characterization and identification. Phenotypic methods were used for putative identification at a generic level based on colony morphology, gram reaction, and microscopy. The result of the gram reaction was used to guide further biochemical tests, including the catalase test, oxidase test, and reaction on triple sugar iron agar (TSI), sulfide-indole-motility (SIM) medium, indole test, methyl red test, Voges Proskauer test, and citrate- IMVC [27–29].

Similarly, the proportion of fecal coliforms (*Escherichia coli*) represented in the TCC was assessed by picking five to ten typical colonies (pink pigmented) from countable VRBA plates and purification by subculturing on MacConkey agar and Eosin methylene blue agar (EMB) plates. Fecal coliform bacteria, such as type 1 *E. coli* formed pink colonies on MacConkey and black colonies with a green metallic sheen on EMB agar plates. The standard IMVIC biochemical tests were used for confirmation when necessary. Isolates that were Indole positive, methyl red positive but negative for Voges Proskauer, and citrate were identified as *E. coli* [27–29].

For the detection of *Salmonella* species, a loop-full sample from 1 : 10 dilution was streaked onto the surface of Salmonella and Shigella agar (SSA). Streaking on the same media was also done after overnight culture of 0.1 mL of a 1 : 10 dilution aliquots into a tube containing 5 mL of sterile nutrient broth. The latter method was done to resuscitate cells that might be in a viable but not cultivable state due to metabolic injury. At the end of the incubation, well-isolated typical colonies (colorless colonies with a black center) were picked and purified by repeated subculturing, and their colony morphology was noted. Presumptive purified isolates were subjected to a biochemical test for confirmation [27–29].

### 2.7. Data Analysis

All data were recorded, and basic statistics for different datasets were analyzed using SPSS version 20. To calculate the average load from multiple plates, the following formula was used [24]:

(1) $$\begin{array}{c} N=\frac{\sum C}{\left(n_{1}+0.1n_{2}\right)d}, \end{array}$$

where ∑*C* is the sum of colonies counted on all the dishes retained; *n*_1_ is the number of dishes retained in the first dilution; *n*_2_ is the number of dishes retained in the second dilution; and *d* is the dilution factor corresponding to the first dilution. Final values were transformed into log_10_ colony forming units per g (log_10_ CFUg^−1^) for ease of manipulation and comparison. Descriptive summary values like average, range, and percentages were used to compare microbial load values within and between the sample types. The paired *T*-test was used to compare the microbial loads of the raw and semicooked fillet samples, and *p* < 0.05 was used to decide on the statistical significance of the observed differences.

## 3. Results and Discussion

### 3.1. Aerobic Mesophilic Bacterial Count (AMBC)

The aerobic mesophilic bacterial counts (AMBC) of the raw/fresh fillet samples ranged from 6.68 to 9log_10_CFUg^−1^with the mean value being 8.53 log_10_CFUg^−1^(Table 1). These values are beyond the recommended acceptable criteria of 5.60–7.00 log_10_ CFUg^−1^ for raw fish fillets [30]. Mean AMBC in raw fish fillets exceeding this value implies gross contamination as a result of poor hygienic conditions of the water source and mishandling during processing. The range of AMBC for the raw fillet samples in this study was in agreement with the 3 × 10^7^ to 4 × 10^9^ CFUg^−1^ of raw fillet samples reported from Khartoum state, Sudan [31]. On the other hand, it was higher than the reported 6.67log_10_CFUg^−1^for samples from Arbaminch, [32], the 6.79 log_10_CFUg^−1^ for samples from Jimma, Ethiopia [33], and the 7.57 log_10_CFUg^−1^ reported for freshwater fish fillets from Latvia [34]. More recently, a similar study for fresh raw fillet samples of different fish species during the hot summer season in Mauritius revealed mean AMBC ranging between 3.1 × 10^5^ CFU/g and 1.4 × 10^7^ CFUg^−1^ [35], which is also lower than the values in the present study.

Considering the semicooked fish fillet samples, the average AMBC ranged from 6.38 to 6.84 log_10_CFUg^−1^ with the mean being 6.67 log_10_CFUg^−1^(Table 1). This value was significantly lower (*P* < 0.05) than that for the raw, fresh fish fillet samples (Table 2). This apparently reflects the effect of cocking temperature and perhaps the lower pH of the semicooked fish sample. The pH is an important intrinsic factor related to fish flesh and influences freshness because it influences bacterial growth [36]. The pH values of semicooked and fresh fish samples ranged from 5.33 to 6.42 and 6.20–6.68, respectively (Table 1). The variations in pH values may be due to physiological conditions, the degree of antemortem activity, or stress [37]. The lower the pH of the fish fillet, the slower is the bacterial growth, and *vice versa* [38]. The higher pH in the raw fillets might be due to higher total volatile bases (TVB) that resulted from the decomposition of nitrogenous compounds by endogenous or microbial enzymes, and this corroborates with the increase in TVB-N [38]. Such an increase in the pH indicates bacterial growth, loss of quality, and incipient spoilage [39].

According to the guidelines by the Institute of Food Science and Technology, London, UK, the average AMBC of heat-treated fish prepared under good manufacturing practice should be less than 4 log units [40]. However, all the semicooked fish samples in the present study showed values above six log units. This finding implies that the initial microbial load in the raw fish fillets was so high that normal cooking temperatures were not sufficient to reduce the load to an acceptable level.

The average AMBC of the semicooked fish samples in this study was slightly greater than the 8.344 × 10^5^−3.108 × 10^6^CFUg^−1^ range of AMBC reported for smoked fish samples [41]. However, it was lower than the 1.5 × 10^5^−1.6 × 10^8^ CFUg^−1^ range of AMBC reported for cooked fish samples sold in Khartoum state, Sudan [31]. The variations among the studies are perhaps reflections of differences in the quality of the source water bodies and the concomitant microbial loads of the initial raw products and handling procedures. How long the raw product was stored before cooking also has a bearing since it is common practice to stock supplies in catering establishments.

According to the International Commission on Microbiological Specifications for Foods [30], when the mean AMBC of fresh raw fillets exceeds the value of 6 log units, it suggests inadequate refrigeration or long storage under refrigeration [40]. It is not uncommon among food caterers to stock supplies of fish fillets in refrigerated storage during times of high production for use in times of scarcity. The mean AMBC of both types of fillet samples in the present study exceeded this level. Likewise, according to Croatian microbiological standards, the maximum permissible AMBC limit for fresh/frozen fish is 3 log units [42]. All the fish samples in the present study showed AMBC levels two-fold higher than this limit. Usually, the muscles and internal organs of healthy fish are sterile [16]. However, various levels of contamination are inevitable during evisceration and processing by either the intestinal or skin flora of the fish, as well as from the fish handler, environment, and utensils.

### 3.2. *Staphylococcus aureus* Count (SC)

The mean *Staphylococcus aureus* counts (SC) of the raw fish fillet samples ranged from not detectable (zero) to 4.3 log_10_CFUg^−1^ with the mean value being 2.85 log_10_CFUg^−1^ (Table 1). This value is lower than the 6.4 log_10_ CFUg^−1^ for raw fish fillet samples sold in Jimma, Ethiopia [33] and the 2.7 × 10^4^CFUg^−1^ - 1.23 × 10^5^CFUg^−1^ values reported for fresh fillets from Mauritius [35]. In the present study, *S. aureus* was detected and counted in 34 (85%) of the raw fillet samples (Table 1). This value is much higher than the 65% prevalence reported for frozen raw fillet samples collected from Lake Abaya and Chamo in Arbaminch [32]. The difference in prevalence might be due to differences in hygienic handling during processing as well as treatment received. *Staphylococcus aureus* is capable of producing enterotoxin and is noted to survive for extended periods in hostile environments [43]. According to recommended guidelines [44], when the count of *S. aureus* in ready-to-consume foods is in excess of 4 log units, the item should be regarded as potentially hazardous. Of the 40 raw fillet samples, 11 (27.5%) showed counts that exceeded this value (Table 1).

In general, *S. aureus* is not considered part of the normal flora of fish [45], and its presence in fish fillets therefore likely reflects contamination due to unhygienic handling practices, cross-contamination from equipment, or processing the environment [46]. The isolation of enterotoxigenic *S. aureus* from raw fish fillets was reported at varying rates elsewhere, including in 6.5% of samples in Egypt [47], in 17.75% in Iraq [48], and in 24.47% of samples in India [49].

In the absence of refrigeration, the initial contaminants can multiply to a sufficient number to cause food poisoning. The presence of *Staphylococcus aureus* in ready-to-eat food items at high counts suggests poor handling and processing and should be considered a public health concern in populations where the consumption of raw fish fillets is rampant [38, 50]. According to a recent survey in the central part of Ethiopia, more than three-fourths (77%) of the consumers preferred consuming raw fish [51].

Concerning the semicooked fish samples, the average SC ranged from not detectable (zero) to 4.2 log_10_CFUg^−1^, with the mean being 2.63 log_10_CFUg^−1^(Table 1). This value was slightly lower than the mean SC of the raw fillet samples. The observed difference in mean SC between the two types of fillet samples was not statistically significant (Table 2). The relatively lower SC in the semicooked fish samples could be due to the combined effect of cooking and the lower pH value. However, it should be noted here that enterotoxin-producing strains of *S. aureus* can grow in a substratum with a pH range of 4 to 10 [52]. As in the case of the raw fillet samples, *S. aureus* was detected and counted in 85% (34 of 40) of the semicooked fillet samples (Table 1). Eight (20%) of the semicooked fillet samples showed hazardous levels of mean SC that exceeded 4 log units (Table 1).

### 3.3. Total Coliform Count (TCC)

The total coliform count (TCC) of raw/fresh fillet samples ranged from not detectable (zero) to 3.2log_10_ CFUg^−1^ with the mean value being 1.51 log_10_CFUg^−1^(Table 1). Coliform bacteria were not detectable in two of the semicooked samples and four of the raw/fresh fillet samples (Table 1). The mean TCC in the present study was lower than the 6.82 log_10_ CFUg^−1^ value reported for fresh fillets sold in Jimma, Ethiopia by Shiferaw et al. [33]. The finding of coliform bacteria like *E. coli* in numbers exceeding 100 per gram in any type of ready-to-consume food is considered to indicate poor sanitary quality [30].

With regard to the semicooked fish fillet samples, the mean TCC ranged from not detectable (zero) to 3.2 log_10_CFUg^−1^ with the mean value being 1.79 log_10_ CFUg^−1^ (Table 1). This value is slightly lower than the mean TCC value for fresh raw fish fillets, although the difference was not statistically significant (Table 2).

### 3.4. Incidence of *Salmonella* Species and *Escherichia coli*

The incidences of *Salmonella* species and *E. coli* in the fresh, raw, and semicooked fillet samples are presented in Table 3. The overall incidences of *Salmonella* species and *E. coli* in the fresh, raw fillet samples were 30% and 35%, respectively. On the other hand, the incidences of the respective bacterial species in the semicooked fillet samples were 25% and 7.5%. The higher incidences of the *Salmonella* species and *E. coli* in raw fresh fillet samples than in the semicooked one is commensurate with expectations since heat treatment, albeit partial, is likely to reduce their survival. The widespread occurrence of members of the *Enterobacteriaceae*, including pathogenic *E. coli* and *Salmonella* species in tropical aquatic environments is well known [46]. The major inland water bodies in Ethiopia, including Lake Hawassa, are situated near major urban areas, where contamination from leaching pit latrines and septic tanks as well as flooding inflows of municipal sewage, is quite common. It is not surprising to encounter *E. coli* and *Salmonella* species in fish sourced from these water bodies. The incidence of *Salmonella* species in the raw fillet samples in the present study was higher than the 7.5% incidence reported by Teka et al. [31] for fresh fillet samples from Arbaminch, southern Ethiopia. Tesfaye et al. [53] also reported the finding of salmonellae in 5.17% of the skin, gills, and intestine of fish samples from Lake Haike in northern parts of Ethiopia, which is lower than the incidence in the present study. Likewise, Ohalete et al. [54] also reported a lower incidence (20%) of *Salmonella* species than that of the present study for fresh fillet samples from Nigeria. On the other hand, Hiko et al. [55] reported the finding of salmonellae in the kidneys and oral cavities of 30 fish samples from Lake Tinike, Eastern Ethiopia. Though the isolation was from specific fish organs, the incidence rate is in concordance with the present study. The observed differences in the incidences among the different studies may reflect differences in the analytical methods, the pollution level of the source lakes, and the sanitary and hygienic condition of the processing by the vendors.

With regard to *E. coli,* the 35% incidence in the raw fresh fillet samples in the present study is lower than the 42.5% incidence reported for frozen Nile tilapia fillet samples from markets in Arbaminch, Ethiopia [31]. On the other hand, Tilahun and Engidawork [56] reported the isolation of *E. coli* from 11 of 131 (8.4%) swab samples from the muscles of different species of fresh fish samples in Hawassa, which is lower than that of the present study. Elsewhere, Rocha et al. [57] reported a 9.09% incidence of *E. coli* from fresh fillet samples in Fortaleza, Brazil. Elsherief et al. [58] also reported a 12% incidence of *E. coli* from fresh fillet samples, which is lower than that of the present study. While there existed differences in the analytical methods used among the studies, the higher incidence of *E. coli* in the fresh fillet samples in this study implies gross pollution of the source lake as well as poor handling, preparation, and storage practices.

*E. coli* is a fecal coliform bacteria that usually originates from the guts of warm-blooded animals and is not part of the normal bacterial flora in fish [57]. Owing to this, *E. coli* is widely used as an indicator of sanitary conditions. The finding of a high incidence and above certain levels per gram is considered to reflect the contamination of fish or the source water with human and animal feces. Trivial observation of the surrounding area of Lake Hawassa, especially at some landing sites reveals open defecation, washing, bathing, and swimming as widespread daily activities along the shoreline and possible seepage of untreated sewage from nearby residential areas due to the absence of a protective buffer zone that separates the lake from drainages and flood. In addition to being an indicator of sanitary quality, several pathovars of *E. coli* are known to cause different types of diseases in humans with varying degrees of severity. Among these, the food-borne pathogenic *E. coli* serotype O157: H7 has been recently reported to be isolated from different species of fish samples from Lake Hawassa [56]. Although this study does not include the determination of serotype, the existence of *E. coli* O157: H7 in Lake Hawassa should be of great concern as it is known to cause hemorrhagic colitis and life-threatening hemolytic uremic syndrome [59–62].

### 3.5. The Dominant Aerobic Mesophilic Bacteria

**Figure 1:**
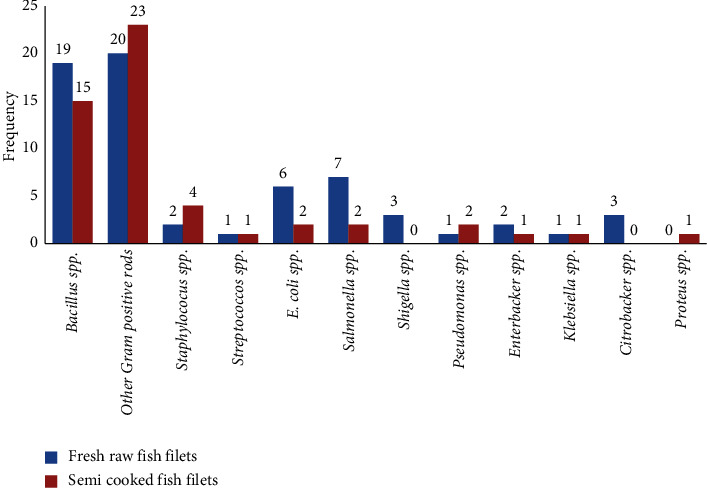
The frequency of the dominant aerobic mesophilic bacterial genera in raw fresh and semicooked Nile tilapia fillet samples marketed in Hawassa, southern Ethiopia, 2018.

A total of 117 aerobic mesophilic bacteria (AMB) were isolated, consisting of 65 from raw, fresh, and 52 from semicooked Nile tilapia fillet samples. Phenotypic characterization, including colony morphology, gram reaction, and a battery of biochemical tests, allowed the putative identification of the majority of the isolates (74/117 or 63.25%) into 11 genera. Of the 65 AMB isolated from the raw fresh fillet samples, 42 (64.62%) were gram-positive bacteria, dominated by isolates related to *Bacillus* species (19/42 or 45.24%) and unidentified, nonspore-forming, gram-positive rod-shaped bacteria (20/42 or 47.62%). *Staphylococci* and *streptococci* constituted the minor genera, each represented by less than five isolates (Figure 1). The remaining 23 AMB isolated from the raw, fresh fillet samples were gram-negative bacteria, dominated by salmonellae (7/23 or 30.43%) and *E. coli* (6/23 or 26.1%). *Citrobacter, Shigella, Enterobacter, Klebsiella,* and *Pseudomonas* species constituted the minor genera each represented by less than five isolates (Figure 1).

Likewise, the majority (43/55 or 78.18%) of the AMB isolates from the semicooked fish samples were gram-positive bacteria, dominated by *Bacillus* species (15/43 or 34.88%) and unidentified, nonspore-forming, gram-positive rods (23/43 or 53.5%). Staphylococci and streptococci constituted the minor gram-positive genera, each represented by less than five isolates. The remaining nine AMB isolated from the semicooked fish samples were gram-negative bacteria, belonging to *E. coli*, *Salmonella,* and *Pseudomonas* species, each represented by two isolates, and *Enterobacter, Klebsiella,* and *Proteus* species, each represented by one isolate (Figure 1).

Several factors affect the number and diversity of microbes found in fish, including the geographical location, the season, and the method of harvest [63]. The bacterial load and diversity of fish in warm tropical lakes are known to be higher and more diverse than those in temperate zones. In addition to the natural flora of the lakes, the fish fillets pick up more bacteria as they proceed in the handling chain from evisceration to cleaning and retail market from handlers, utensils, and the environment. The widespread open defecation of local residents and the unrestricted access that cattle have to freshwater lakes and reservoirs, as well as the exposure of the water bodies to contaminated water run-off, are all potential causes of contamination of the habitats used naturally for the production of fish. In one recent study, it was reported that there is a dramatic worsening of pollution levels in the Lake Hawassa Watershed due to urbanization, the usage of fertilizers in agricultural lands in the suburbs, effluents from industrial facilities, excessive usage of detergents in domestic and industrial facilities, soil erosion, increased sewage pollution, the practice of open defecation and urban runoff [64].

The result of this study revealed that common pathogenic bacteria were associated with fresh and semicooked fillet samples. In a similar study carried out by Moshood and TengkuHaziyamin [65], *Bacillus subtilis, Staphylococcus aureus, Proteus mirabilis, Klebsiella* sp., *Salmonella typhi,* and *Streptococcus* sp. were all found to be associated with smoked and raw fish. The presence of *Staphylococcus aureus* was attributed to the contamination of the fish samples by humans through handling and processing. *Staphylococcus aureus* is capable of producing heat-stable enterotoxin and is noted to survive for extended periods in hostile environments [50]. Consumption of food that contains a high number of enterotoxigenic strains could cause gastroenteritis in susceptible individuals. The presence of *Salmonella* species in semicooked fish indicates poor hygiene handling practices and postprocessing contamination [66].

### 3.6. Food Handling and Hygienic Practices

Food handling and processing are among the main sources of food contamination through poor hygienic practices. In this regard, all food handlers have a basic responsibility to have a high degree of personal hygiene and safe handling practices. However, the result of this study showed that 63.33% of the vendors wash their hands using clean water and soap, but 36.67% wash their hands only with water. Besides this, none of the vendors use antiseptics or disinfectants (Table 4). The nails of 43.33% of the fish handlers in this study were not shortly trimmed and cleaned. This finding is higher than the result of Teklemariam et al. [67] who found that 21.2% of food handlers in Hawassa city hotels did not trim their nails, but lower than the 46.5% reported in the study carried out in Arbaminch, southern Ethiopia [31].

Moreover, 43.34% of the vendors did not cover their hair during food preparation. The present result is consistent with that of Kumie et al. [68] and Wendwesen et al. [31] who reported that 60% and 55.8% of the participants in Ziway and Arbaminch towns, respectively, did not cover their hair during the preparation of food. On the other hand, the proportion of fish vendors who did not cover their hair during food handling in the present study was lower than the reported 98.2% for vendors from Hawassa in a previous study [67].

Remarkably, 63.33% of the respondents cleaned their utensils with water and soap, while 36.67% washed their utensils regularly with water only. In addition, the survey showed that 33.3% of the fish handlers did not have separate work clothes for food preparation, and only 46.66% of them washed their work clothes regularly (Table 4). These findings are concordant with those reported by Teka et al. [31], where only 32.6% of participants washed their clothes. Work clothes should be kept clean and stored separately from private clothes. Otherwise, there is a risk of bringing various types of contaminants, including pathogens, to the fish handling locations, enhancing the risk of cross-contamination. Poor socioeconomic conditions, a lack of infrastructure, and ignorance are among the major challenges to fishery management problems in Ethiopia [18].

According to Adams and Moss [69], providing training for food handlers regarding the basic concepts and requirements of personal hygiene and sanitary handling of food plays an integral part in ensuring a safe product for the consumer. However, the result of this study showed that 96.66% of respondents had poor awareness of the consequences of microbially contaminated fish, and none of the fish vendors had professional training related to the safe handling and processing of fish fillets. Circumstantial observations on the lakeside landing sites showed that most of the fisherman handled and processed fish in unhygienic conditions near the lake shore. They were all performing the task without access to basic processing equipment, such as a clean, room or area, clean water, a facility for washing and disinfecting equipment, a place to wash one's hands, or a facility for handling products. All fish were frequently processed with a single knife and the same cutting board, with infrequent washing and no disinfection in between. It is not uncommon to see some fishermen manually eviscerating fishes, removing the skin, and washing them with lake water.

Although there are some policies and legislation governing the fisheries sector in Ethiopia, they are poorly implemented. The federal fisheries Proclamation No. 315/2003 for Fisheries Development and Utilization [70] was aimed to “conserve fish biodiversity and its environment” as well as prevent and control overexploitation of the fisheries resource, increase the supply of safe and good-quality fish, ensure a sustainable contribution of the fisheries toward food security, and expand the development of aquaculture.” This most recent legal document that is specifically related to the fisheries sector has these goals. Ethiopia has also endorsed the FAO Code of Conduct Article II 1998s which states that member states should adopt appropriate measures to ensure the right of consumers to safe, wholesome, and unadulterated fish and fishery products. In the absence of vigilance in the enforcement of safety and quality standards in fishery production and supply chains, periodic cross-sectional studies like this may serve as early warning alarms [71].

## 4. Conclusion

This study has revealed that both fresh raw and semicooked fish fillets marketed in Hawassa had a higher microbial load than recommended levels. The mean microbial load of the fresh raw fillet samples was significantly higher than that of the semicooked fillet samples. This finding implies that the microbial load in the raw fillets was so high that the normal cooking temperature was not sufficient to reduce the load in the ready-to-eat item to an acceptable level. The aerobic mesophilic bacterial flora of both types of samples was dominated by genera known to be food-borne pathogenic bacteria. *Staphylococcus aureus and Escherichia coli* are potential toxigenic pathogens and are known to cause food poisoning. The detection of *Salmonella and Shigella* in fresh fish samples should be of great public health concern in communities where the consumption of raw fillets is rampant since they are known to cause life-threatening infections. Several factors may be speculated to contribute to the contamination of fish fillets, including poor sanitary quality of the water in the source lake, unhygienic processing, improper storage, transportation, poor handling during preparation, and display for sale. The survey data on the level of awareness and hygienic practice of fish vendors and handlers revealed a lack of training on the proper handling and processing of food, poor personal hygiene of vendors, and unhygienic surroundings could be possible factors for the observed problems.

## Figures and Tables

**Table 1:** The microbial load in log_10_CFUg^−1^ and pH of fresh raw fish fillets and semicooked fish fillets from different sites in Hawassa city, southern Ethiopia, 2018.

SN	Fresh raw fish fillets				Semi cooked fish fillets			
	AMBC	TCC	SC	pH	AMBC	TCC	SC	pH
1	8.19	0	2.69	6.03	6.58	0	4.14	5.67
2	9	0	0	5.61	6.83	0	0	5.46
3	8.19	0	2.62	6.46	6.81	0	4.2	5.46
4	8.18	0	2.66	6.69	6.84	0	0	5.7
5	8.46	0	2.99	6.77	6.78	3.1	3.64	5.21
6	9	0	2.99	6.81	6.6	2.62	4.13	5.36
7	8.14	0	2.86	6.62	6.73	2.72	2.66	5.42
8	8.12	0	3.05	6.13	6.71	3.11	4.13	5.32
9	9	0	3.08	6.69	6.63	2.54	0	5.03
10	8.29	0	2.9	6.68	6.79	2.58	3.63	5.84
11	8.27	0	3.1	6.33	6.74	2.56	0	5.44
12	8.28	0	2.9	6.81	6.71	2.56	4.12	5.93
13	8.45	3.2	3.1	6.47	6.67	3.08	2.59	6.47
14	9	3.14	2.9	6	6.64	2.09	4.15	6
15	8.39	0	3.1	6.65	6.63	2.48	2.66	6.65
16	8.42	3.09	3.26	6.37	6.83	2.8	2.62	6.37
17	9	0	3.1	6.71	6.75	0	0	5.33
18	9	0	3.03	6.25	6.71	0	4.12	5.68
19	8.32	0	3.03	6.02	6.62	0	2.59	5.69
20	8.3	0	2.18	6.3	6.59	0	4.15	5.61
21	8.35	2.9	4.13	6.86	6.47	2.81	3.11	5.81
22	8.42	2.74	4.13	6.23	6.77	0	2.56	5.77
23	8.39	0	0	6.98	6.71	3.15	2.6	5.62
24	8.45	2.64	4.13	6.66	6.8	0	2.8	5.03
25	8.67	2.56	0	6.09	6.47	3.2	2.56	6.4
26	8.67	2.48	3.81	6.7	6.77	2.67	2.48	6.47
27	8.63	2.53	4.14	6.21	6.5	2.51	2.54	6.77
28	6.68	2.54	2.65	6.36	6.84	2.6	2.71	6.03
29	8.77	2.8	2.66	6.42	6.77	2.6	2.53	5.89
30	8.61	2.69	4.12	6.41	6.62	3.09	2.68	5.87
31	8.48	2.51	3.78	6.32	6.48	2.47	0	5.7
32	8.67	2.67	0	6.36	6.67	2.89	2.5	5.36
33	8.47	2.67	0	6.03	6.47	3.1	2.8	5.86
34	8.77	2.47	4.14	6.84	6.77	2.62	3.31	5.23
35	8.5	3.11	0	6.93	6.5	2.72	3.08	5.98
36	8.84	2.67	4.14	6.4	6.84	3.11	3.08	5.81
37	9	2.47	4.3	6.47	6.66	0	2.62	5.33
38	9	3.09	4.13	6.03	6.38	0	2.72	5.68
39	9	2.6	4.16	6.77	6.61	0	2.49	5.76
40	9	2.89	4.16	6.81	6.63	0	2.56	4.61
Mean	8.53	1.51	2.85	6.46	6.67	1.79	2.63	5.72
Min	6.68	0	0	5.61	6.38	0	0	4.61
Max	9	3.2	4.3	6.98	6.84	3.2	4.2	6.77
Std	0.42	1.40	1.34	0.32	0.12	1.35	1.27	0.46

SN = sample number; AMBC = aerobic mesophilic bacterial count; TCC = total coliform count; CS = count of staphylococci; Min = minimum; Max = maximum; Std = standard deviation.

**Table 2:** Comparison of the mean microbial load (AMBC, TCC, SC) and pH values based on the student's *T*-test for the fresh raw fish fillets (*n* = 40) and semicooked fish fillets (*n* = 40) in Hawassa, southern Ethiopia, 2018.

Microbial load parameters	*T*	D*f*	Significance. (2-tailed)	Mean differ	Std. error difference	95% confidence interval of difference	
						Lower	Upper
AMBC	−26.7	45.6	0.000^*∗*^	−1.86	0.07	−2.002	−1.72
TCC	0.92	78	0.360	0.283	0.31	−0.329	0.89
SC	−0.76	78	0.451	−0.222	0.293	−0.804	0.36
pH	−8.5	78	0.000∗	−0.742	0.088	−0.916	−0.57

AMBC = aerobic mesophilic bacterial count; TCC = total coliform count; SC = staphylococcal count; d*f* = degree of freedom; *T* = student's *T*-test, ^*∗*^ = indicates the observed differences in mean microbial load of fish fillets samples for the respective parameters between fresh raw fish fillets and semicooked fish fillets is statistically significant by an independent *t*-test, at *p* value <0.05.

**Table 3:** Frequency of detection of *Salmonella and E. coli* from raw and undercooked Nile tilapia fillets.

Vendor	Raw/fresh fillets (*n* = 40)		Semicooked fillets (*n* = 40)	
	*Salmonella* spp.	*E. coli*	*Salmonella* spp.	*E. coli*
A	1	2	2	1
B	1	1	2	0
C	1	2	3	1
D	1	2	2	1
E	1	1	1	0
F	1	1	0	0
G	2	1	0	0
H	1	1	0	0
I	1	1	0	0
J	2	2	0	0
Total (%)	12 (30%)	14 (35%)	10 (25%)	3. (7.5%)

**Table 4:** Awareness level regarding microbial contaminants, hygiene in preparation, and food safety among fish handlers (*n* = 30) in Hawassa city.

Variables		Number of representatives	Percent (%)
Frequent hand washing habit	Using clean water	11	36.67
	Clean water and soap	19	63.33
	Clean water, soap, and antiseptics	0	0.00

Hair covering habit	Yes	13	43.34
	No	17	56.66

Frequent nail-trimming habit	Yes	17	56.66
	No	13	43.33

Habit of washing work clothes	Yes	14	46.66
	No	16	53.33

Use of separate work clothes	Yes	20	66.66
	No	10	33.33

Method of washing utensils	Only water	11	36.67
	Water with soap	19	63.33
	Water with soap and detergent	0	0.00

Awareness about microbial contamination	Yes	5	16.66
	No	25	83.33

Training in food hygiene and safety	Yes	0	0.00
	No	30	100

## Data Availability

All supporting data for this work are included in the main manuscript. The original data generated and/ or analyzed during this work can be obtained from the corresponding author on reasonable request.
